# Over my fake body: body ownership illusions for studying the multisensory basis of own-body perception

**DOI:** 10.3389/fnhum.2015.00141

**Published:** 2015-03-24

**Authors:** Konstantina Kilteni, Antonella Maselli, Konrad P. Kording, Mel Slater

**Affiliations:** ^1^Event Lab, Department of Personality, Evaluation and Psychological Treatment, University of BarcelonaBarcelona, Spain; ^2^IR3C Institute for Brain, Cognition, and Behaviour, University of BarcelonaBarcelona, Spain; ^3^Sensory Motor Performance Program, Rehabilitation Institute of ChicagoChicago, IL, USA; ^4^Department of Physical Medicine and Rehabilitation, Northwestern UniversityChicago, IL, USA; ^5^Department of Physiology, Northwestern UniversityChicago, IL, USA; ^6^Institució Catalana de Recerca i Estudis Avançats, Passeig Lluís Companys 23Barcelona, Spain

**Keywords:** body ownership, rubber hand illusion, multisensory perception, body semantics, causal inference

## Abstract

Which is *my* body and how do I distinguish it from the bodies of others, or from objects in the surrounding environment? The perception of our own body and more particularly our sense of body ownership is taken for granted. Nevertheless, experimental findings from body ownership illusions (BOIs), show that under specific multisensory conditions, we can experience artificial body parts or fake bodies as our own body parts or body, respectively. The aim of the present paper is to discuss how and why BOIs are induced. We review several experimental findings concerning the spatial, temporal, and semantic principles of crossmodal stimuli that have been applied to induce BOIs. On the basis of these principles, we discuss theoretical approaches concerning the underlying mechanism of BOIs. We propose a conceptualization based on Bayesian causal inference for addressing how our nervous system could infer whether an object belongs to our own body, using multisensory, sensorimotor, and semantic information, and we discuss how this can account for several experimental findings. Finally, we point to neural network models as an implementational framework within which the computational problem behind BOIs could be addressed in the future.

## Introduction

*“I swear to God, cross my heart, I haven't (been kidding). A man should know his own body, what's his and what's not—but this leg, this thing…. doesn't feel right, doesn't feel real—and it doesn't look part of me”* (Sacks, 1985).

There would be nothing wrong or weird with the above statement, if we thought that the person speaking had been referring to a fake leg, which for some unknown reason was placed close to his body. However, in fact the quote is from a brain-damaged patient talking with his doctor about his own paralyzed leg. The bizarre neurological syndrome of somatoparaphrenia reveals that our ability to recognize our own body parts can dramatically deteriorate in certain brain damage conditions (Vallar and Ronchi, 2009; Feinberg et al., 2010). Astonishing as it is, the case of somatoparaphrenia gives prominence to the multidisciplinary research topic (Gallagher, 2000; Jeannerod, 2003; Blanke and Metzinger, 2009; de Vignemont, 2011) that aims to address a seemingly trivial question: how does our brain distinguish between our *own* body and those of other people or objects?

A key difference between the perception of our own body and that of others' bodies or objects in the environment lies in the type of sensory input available to the brain. In processing our own body, the brain has access to a set of sensory information—such as somatosensation, thermosensation, nociception, interoception, and vestibular signals—that is not available for the perception of other objects or bodies. Yet, evidence from somatoparaphrenic patients suggests that the intact sensory processing from separate modalities may not be in itself sufficient for the emergence of the feeling of body ownership. Indeed, it has been proposed that somatoparaphrenia may be due to an impairment in processing multisensory signals (Vallar and Ronchi, 2009). Therefore, the sense of body ownership should be regarded as the outcome of the brain's processes that integrate different sensory cues into the unified perception of “my body.”

Another key difference concerns the fact that in own-body perception all the sensory and motor cues that converge into the “my body” percept are strictly bound by physical laws. For example, when striking a fist on a table, the view of the contact is always accompanied by the punching tactile sensation in our hand. This is not the case for the perception of external objects or others' bodies that, although multisensory in nature, is not subject to such strict constraints. Just consider this example: you see a dog in a park and you hear a barking sound at about the same moment and coming from the same direction. Even if it is probable that it was the seen dog that was barking, there is also the possibility that there is a second dog just behind the tree. Thus, while in the perception of external events we can contemplate the possibility of different sources, in the perception of “my body” this is not the case, since all multimodal cues involved originate from the same source: the physical body.

Due to the fact that the body-related multimodal cues are tightly bound together and not independent, it is difficult to experimentally investigate body ownership with the methods adopted in multisensory research. In contrast, when studying the multisensory perception of external objects, including others' bodies, experiments typically involve the concurrent presentation of sensory signals that are independent. This permits the introduction of delays between the occurrences of the stimuli, to present them from different positions, or even to manipulate their information content so that these may refer to the same context or not. Therefore, it is possible to investigate how multisensory perception is influenced by the spatial, temporal, and semantic relationships between the manipulated stimuli (Doehrmann and Naumer, 2008; Alais et al., 2010). The same methods cannot be directly applied to study body ownership. For example, it is not possible to introduce a temporal delay between seeing our fist striking the table and feeling the punching sensation in the hand, unless through the use of devices such as cameras and displays.

Given the limitations inherent in the study of the physical body, experimental research has been extensively conducted through exploiting the illusions of body ownership. In these illusions, healthy adults experience non-bodily objects (e.g., artificial limbs) as belonging to their own body, when presented with crossmodal stimuli applied to the hidden real body part and its fake counterpart. Due to the fact that the stimuli have two independent sources (i.e., the real and the fake body part), experimenters have been able to flexibly manipulate their spatial, temporal, and semantic relationships. In this way, body ownership illusions (BOIs) therefore offer a powerful experimental tool to examine how the sense of body ownership emerges from multisensory processing operated by the brain.

Our understanding of how the brain builds the sense of body ownership can benefit from identifying the basic principles that govern the induction of body ownership illusions and formulating the latter within a computational framework. Consequently, the aim of this paper is two-fold. First, to review the experimental literature, investigating how the spatial, temporal, and semantic congruencies of the tested crossmodal stimuli contribute to eliciting body ownership illusions. Second, to discuss and propose different theoretical accounts that could possibly cast the experimental findings into a unifying computational context. We start by introducing body ownership illusions and distinguishing these from other classes of body illusions that, together with the former, have provided essential insights on how the own-body percept is built through multisensory and sensorimotor information. Following this, we present the main results of experimental work on body ownership illusions, classifying them on the basis of the crossmodal triggers and semantic information that these have manipulated. This classification is functional to the discussion of different theoretical accounts of body ownership.

## Body illusions to study own-body perception

Body illusions refer to those psychological phenomena in which the perception of one's own body importantly deviates from the configuration of the physical one, e.g., in terms of size, location, or ownership. Since their induction is achieved through multisensory and/or sensorimotor stimulation, body illusions provide essential insights on how the own-body percept is built in real-time, on the basis of the stimuli that are currently available to the brain.

**Figure 1:**
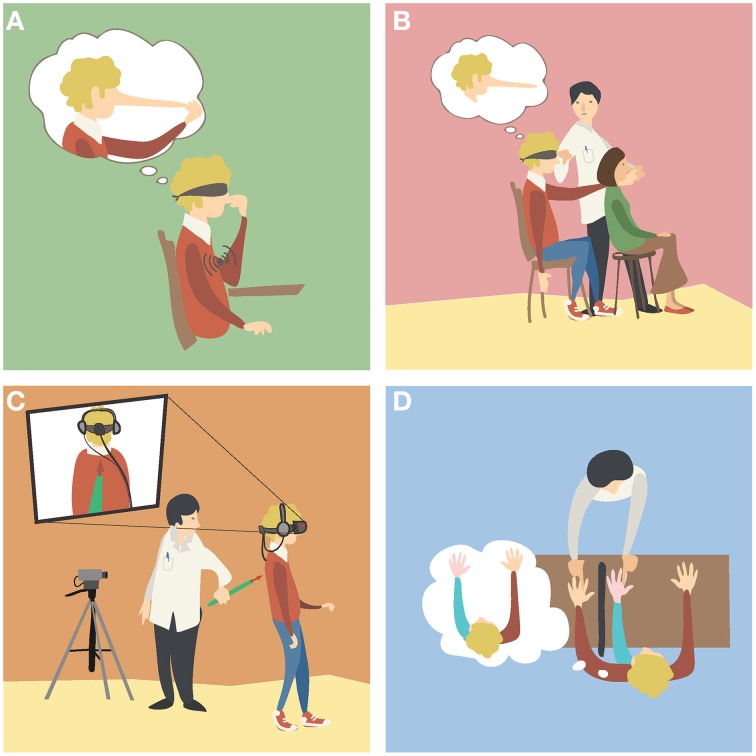
**Examples of body illusions. (A)** *The Pinocchio illusion*. A blindfolded participant receives vibration on his biceps while touching the tip of his nose with his fingers. The illusory extension of the arm (Goodwin et al., 1972) generates the illusion that his nose, his fingers or both are elongating (Lackner, 1988). **(B)** *The phantom nose illusion*. The experimenter moves the finger of a blindfolded participant to tap the nose of another subject, while simultaneously tapping the nose of the participant. As the participant's movements and his finger contact with the other subject's nose are synchronous with the touch he receives on his nose, the participant experiences the illusion of tapping his very long nose (Ramachandran and Hirstein, 1998). **(C)** *An out of body illusion*. The participant sees a video of his back as if he were located behind it. The experimenter touches the back of the participant with a stick while the participant sees it online in the video. As the seen and the felt stimulation is synchronous, the participant perceives illusory drifts in his self-location toward the seen body (Lenggenhager et al., 2007). **(D)** *The rubber hand illusion*. The participant sees a rubber hand placed in front of him, while his real hand is concealed from view. The experimenter strokes both hands at the same time, and after some time the participant perceives the fake hand as if it were his own hand (Botvinick and Cohen, 1998).

A representative example of body illusions are the *body distortions illusions*, in which people can perceive that the size or the posture of their body part(s) have changed dramatically without necessarily satisfying the anatomical constraints of the human body. A method to induce illusory body distortions relies on kinaesthetic illusions, in which blindfolded subjects experience the illusory movement of a static body part and therefore non-veridical proprioceptive states, when the tendon muscle of a physically constrained joint is mechanically vibrated (Goodwin et al., 1972). Importantly, the illusory motion of the stationary body part can capture other non-movable body parts it is in contact with, yielding the impression that these change in size (Lackner, 1988; de Vignemont et al., 2005; Ehrsson et al., 2005b). Similar distortion illusions have been shown to occur for numerous other body parts (Lackner, 1988), to correlate with activation of areas in the lateral parietal cortex (Ehrsson et al., 2005b) and to modulate the tactile processing on the body part perceived as distorted (de Vignemont et al., 2005). A well-known example is the Pinocchio illusion (Figure 1A). Alternatively, a distortion illusion similar in phenomenology can be induced through temporal correlations between undistorted proprioceptive information and tactile input, as for example the phantom nose illusion (Ramachandran and Hirstein, 1998) (Figure 1B). Independently of the employed methodology, the body distortion illusions demonstrate that the brain computes the perceived body posture and shape dynamically and in a flexible fashion, without the need to satisfy the anatomical constrains of the human body.

Another remarkable class of body illusions are the experimentally induced *out of body experiences*, in which people perceive their self to be dislocated from the spatial origin of their visual perspective (Lenggenhager et al., 2007), and/or of looking at their body from a distance (Ehrsson, 2007). One of the first reports of these illusions was attained through the use of a mirror device and goes back to the end of the nineteenth century (Stratton, 1896). In the recent experimental settings participants wear a head mounted display (HMD), which is fed by a camera that records the back of the participants from distance. Thus, participants see their body from the back, i.e., from a third person visual perspective (3 PP), in contrast with the first person visual perspective (1 PP) that we normally have on our body when looking downward. Through applying tactile stimulation on the participants' back while they see the instrument touching their back in the online video, participants experience illusory drifts in their self-location toward the body seen in the video (e.g., Lenggenhager et al., 2007) (Figure 1C). Illusory changes in self-location were also reported when subjects were tapped on their chest with a stick while another stick was synchronously waived underneath the cameras (e.g., Ehrsson, 2007). Analogous illusory experiences were reported by subjects standing between two mirrors that face each other, while stroking their cheek and watching the several reflections of their bodies (Altschuler and Ramachandran, 2007). Systematic experimental findings from the out of body illusions demonstrate that the perceived location of the self can be detached from that of the physical body, similar to reports from patients undergoing out-of-body experiences of neurological origin (Blanke and Mohr, 2005). This class of illusions provides therefore an important benchmark for studying the role of multisensory integration in the sense of self-location and self-consciousness (Blanke and Metzinger, 2009; Blanke, 2012).

### Body ownership illusions

In distinction to body distortion and out of body illusions, *Body ownership illusions* refer to the illusory perception of non-bodily objects (e.g., artificial limbs) as being parts of one's own body and as being the source of the associated bodily sensations, such as touch. For example, one can get to experience a mannequin's hand as his/her own hand, and have strong physiological responses when seeing it being attacked with a knife (Ehrsson et al., 2007).

One of the earliest versions of this class of illusion was reported by Tastevin (1937), who described how people could perceive an artificial finger protruding from a cloth as their own finger, when the latter was hidden from view. Sixty years later, Botvinick and Cohen (1998) provided the first report of the rubber hand illusion (RHI), in which healthy adults experience a rubber hand as if it were their own hand. In this experimental setup, subjects have their hand concealed from view, while they see a rubber hand of the same laterality, placed in a similar posture. The experimenter strokes both rubber and real hands simultaneously, and after some time the majority of participants report perceiving the touch as if coming from the rubber hand, and the latter as being part of their own body (Figure 1D). In addition, when asked to point blindly to the position of their left hand, participants typically give proprioceptive estimations that are shifted toward the rubber hand compared to their estimations before the stimulation (e.g., Botvinick and Cohen, 1998; Tsakiris and Haggard, 2005).

Apart from proprioceptive drifts, the RHI has been shown to decrease the temperature and slow down the processing of tactile input from the real hand (Moseley et al., 2008; Hohwy and Paton, 2010) and to trigger the participants' autonomic responses when seeing the rubber hand under threat (e.g., Armel and Ramachandran, 2003). Interestingly, it has been shown that brain areas associated with anxiety and interoceptive awareness selectively activate when, during the RHI, the fake limb is under threat and at a similar level as when the real hand is threatened (Ehrsson et al., 2007). Moreover, the subjective reports about the intensity of the illusion, typically addressed through questionnaires, have been shown to correlate positively with many of the objective measures mentioned above—e.g., proprioceptive drifts (Longo et al., 2008b), brain activity (Ehrsson et al., 2004; Limanowski et al., 2013), and decrease in temperature of the real counterpart (Moseley et al., 2008).

Beside correlated visuotactile cues, the RHI has been demonstrated also under different multimodal stimuli. For example, the illusion has been induced with correlated visual and motor information, that is when participants are performing movements with their unseen body part while seeing the fake counterpart moving similarly (e.g., Dummer et al., 2009; Kalckert and Ehrsson, 2012, 2014a,b). In addition, just seeing the rubber hand at the same position of the occluded real hand, that is in the mere presence of coincident visual and proprioceptive information, can elicit the illusion of body ownership (Giummarra et al., 2010).

The illusions of body ownership have been also induced toward full humanoid bodies, by using HMDs that permit seeing the fake body in the same spatial location as the real body (Petkova and Ehrsson, 2008; Slater et al., 2010; Petkova et al., 2011b; Maselli and Slater, 2013). These full body illusions were induced through visuotactile (e.g., Petkova and Ehrsson, 2008), visuomotor (e.g., Peck et al., 2013), and visuoproprioceptive information (Maselli and Slater, 2014). Moreover, they were shown to have physiological and proprioceptive correlates analogous to those of the RHI (Petkova and Ehrsson, 2008; Llobera et al., 2013; Maselli and Slater, 2013, 2014).

The induction of these illusions was shown even in the absence of any visual input. In the so-called “somatic RHI,” blindfolded participants have their left index finger passively moved by the experimenter to touch a rubber hand, whereas the experimenter synchronously touches their right hand. After some seconds of correlated tactile and proprioceptive information, the participants typically report the illusion of touching their own hand, instead of the rubber one (Ehrsson et al., 2005a). Although using a different methodology, the somatic RHI was found to correlate selectively with enhanced activity in the premotor cortex, as in the classic RHI and the full body ownership illusion (Ehrsson et al., 2004, 2005a; Petkova et al., 2011a). This remarkable convergence of results supports the view that body ownership is not determined by the type of sensory triggers employed, but emerges from the synergetic processing of the multimodal information available at a specific time.

Extending the findings from other bodily illusions, body ownership illusions reveal that our brain dynamically computes which are our own body parts on the basis of the available multisensory and sensorimotor information. Nevertheless, although the numerous studies bring along a wealth of insights about the multisensory mechanisms that underlie own-body perception, little emphasis has been devoted to casting this information into a coherent and comprehensive picture. In the following, we review the literature on body ownership illusions with the aim of identifying the multimodal triggers and constraints that govern them. Given the different response variables used by different experimenters, our criterion about the impact of the manipulation of the experimental variable in eliciting the illusion is primarily based on the most common measure, the subjective scores collected through questionnaires. However, we also refer to other objective measures especially in the cases where no questionnaires were administered. Moreover, given the extensive literature, we limit our scope to the body ownership illusions where visual information was available.

## Multimodal triggers and semantic constraints in body ownership illusions

**Figure 2:**
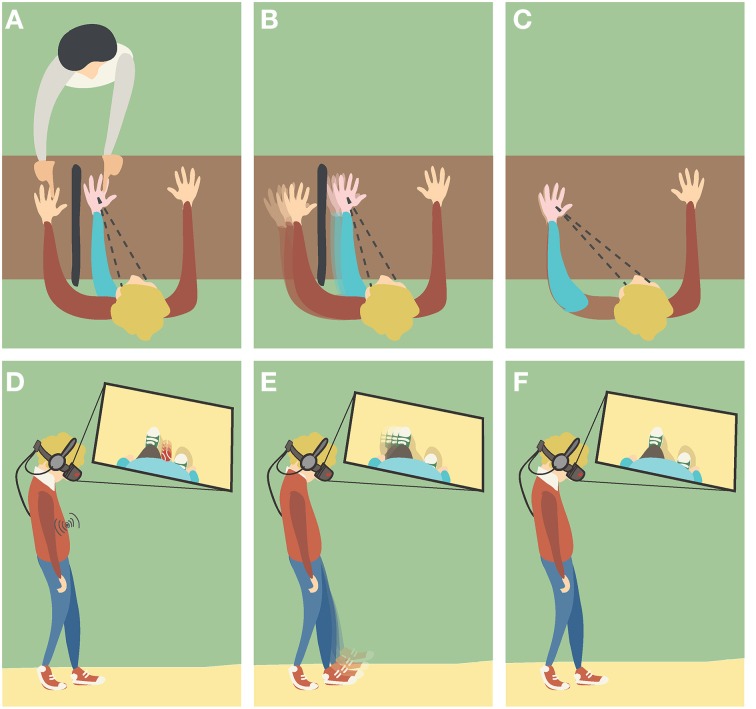
**Different induction methods of Body Ownership Illusions (BOIs). (A,D)** *Visuotactile triggers:* the participant is watching the fake hand/body placed in a plausible posture and being touched, while receiving synchronous tactile stimulation in the real counterpart that remains out of view. **(B,E)** *Visuomotor triggers*: the participant is performing movements with his real hand/body that remains out of view, while watching the fake counterpart moving synchronously. **(C,F)** *Visuoproprioceptive triggers*: the participant is watching the fake hand/body placed in an overlapping position with the real counterpart that remains out of view.

The elicitation of body ownership illusions (BOIs) has been shown under different experimental setups that provide different crossmodal stimuli (Figure 2). In this section, we present several experimental studies on BOIs by classifying them in terms of the main crossmodal stimuli provided (i.e., visual and tactile, visual and proprioceptive, and visual and motor), or of the semantic feature of the fake body that these have manipulated. With the specific aim to highlight the principles that permit the induction of BOIs, we investigate the importance of spatial and temporal correspondence for each pair of crossmodal stimuli, as well as the role of semantic information that the view of the non-bodily objects brings along. To provide a link between the illusions of body ownership and our normal sense of body ownership, for each of considered the components we present a brief overview of its known role in our own-body perception.

### Visuotactile triggers for body ownership illusions

#### Visuotactile integration in own-body perception

The experience of our own body is importantly shaped by visual and somatosensory signals. One of the main contributions of visuotactile integration concerns the information about contact events between our body and the surrounding environment. For example, when a fly lies on our hand, both vision and touch inform the brain about the time and the location of the contact. But even before the physical contact occurs, vision can provide anticipatory information about where and when the fly is probably going to land, which allows us to take actions accordingly.

Beside its established role in determining the shape and texture of objects we explore with our hands (Ernst and Banks, 2002; Lacey et al., 2010; van Beers et al., 2011; Helbig et al., 2012), visuotactile integration is considered to be critical in perceiving our body and the space nearby. This was first suggested by the discovery of visuotactile neurons in the brain of monkeys: the visual receptive fields of these cells extends outwards from the mapped body part into the external space and shifts along with body movement (Rizzolatti et al., 1981; Graziano and Gross, 1993; Duhamel et al., 1998). Because of these properties, these bimodal neurons were proposed to build an enlarged and flexible representation of the space surrounding the body—the peripersonal space—that mainly serves to guide our movements (Graziano and Gross, 1994; Fogassi et al., 1996; Graziano and Cooke, 2006).

Homologous visuotactile populations have been identified in the human brain with analogous properties and functionality (Lloyd et al., 2003; Makin et al., 2007; Gentile et al., 2011; Sereno and Huang, 2014). In addition, the importance of visuotactile integration in the spatial representation of the body, has been largely supported by behavioral studies (Spence et al., 2004).

Altogether, experimental evidence from monkey neurophysiology to human behavior indicates the fundamental role of visuotactile populations in flexibly defining the dynamics of our own body spatial boundaries.

#### Methods and apparatus for visuotactile stimulation in BOIs

Turning to BOIs, the first report of the RHI was induced through applying tactile stimuli on the occluded real hand and the visible fake counterpart (Botvinick and Cohen, 1998). The procedure was quickly established as a standard protocol and extended to full BOIs as well (Figures 2A,D).

With respect to the employed methods, seen and felt touch have been typically delivered manually by the experimenter (e.g., Botvinick and Cohen, 1998; Armel and Ramachandran, 2003; Petkova and Ehrsson, 2008). Alternative setups used cameras, screens or virtual reality methods. By these technological means, some experimenters manually stimulated only the real body (or body part), whilst the participants watched a video stream of the real body part being touched (e.g., Tsakiris et al., 2006) or a virtual object touching an artificial body (or body part) seen in stereo mode (e.g., Slater et al., 2008). Alternatively, computer-controled administration of touch was implemented by programming robot arms or stepper motors that touched both the real and the fake body (or body parts) (Tsakiris et al., 2008; Rohde et al., 2011), or by attaching mechanical vibrators on the real body and programming them to fire when the participants watched the virtual body being touched through the HMD (Pabon et al., 2010; Evans and Blanke, 2013; Maselli and Slater, 2013).

#### Spatial and temporal principles of visuotactile integration as trigger of BOIs

Systematic experimental evidence has shown that the RHI is induced when the real hand and the fake counterpart are touched at the same time and at homologous regions in hand-centered coordinates. For example, when spatiotemporal mismatches were introduced between the seen and felt touch, the RHI was significantly inhibited (Armel and Ramachandran, 2003; Ehrsson et al., 2004; Slater et al., 2008). Similar results were found for full BOIs toward plastic mannequins seen from a 1 PP; participants perceived the mannequin's body as their own body when the real and the fake abdomen part were touched synchronously, but not when asynchronously (Petkova and Ehrsson, 2008). Nevertheless, other studies have shown that when the fake body (or body part) is realistic and has overlaps in space with the real body counterpart, positive scores of ownership can be reported even in presence of asynchronous visuotactile stimulation (Longo et al., 2008a; Maselli and Slater, 2013).

A systematic study on the importance of temporal alignment revealed that, when delays smaller than 300 ms between the stimulations were introduced, participants perceived the touch on their hand as if caused by the stimulation applied to the rubber hand, while for larger delays these illusory feelings were significantly attenuated (Shimada et al., 2009, 2014). In addition to the temporal coupling, spatial congruence was also found to be essential for the induction of the illusion. Stroking the rubber and the real hands in temporal synchrony but at different locations (e.g., index vs. little fingers, palm vs. forearm, or index vs. middle finger) abolished the illusion (Kammers et al., 2009; Limanowski et al., 2013; Riemer et al., 2014), demonstrating that temporal matching alone is not sufficient for the RHI. Similar were the findings on proprioceptive drifts when a spatial mismatch was introduced between the seen and the felt synchronous strokes (Costantini and Haggard, 2007).

### Summary

Overall, the experimental results suggest that the induction of BOIs depends critically on the spatiotemporal congruence between the seen and the felt stimulation, which is a sufficient condition to induce the illusions. However, the fact that BOIs could occur in presence of visuotactile asynchronies when the fake body is seen superimposed onto its real counterpart, indicates that visuotactile correlations are not a necessary prerequisite for the illusion under such configuration.

### Visuomotor triggers for body ownership illusions

#### Visuomotor integration in own-body perception

Our own-body perception is highly determined by our movements since these provide information that facilitates self-other distinction. For example, when a pianist plays the piano in a duet, she uses—amongst others—her motor information together with visual, tactile and proprioceptive feedback to recognize which of the two right hands she sees is her own.

The experience of moving and acting in space involves an extremely rich content of body related information that goes far beyond the multisensory integration of two or more sensory modalities. This can be better understood considering the distinction between active vs. passive movements. In a passive movement there is no motor intention: an externally generated force displaces our body (body part) and consequently, a number of sensory information, including vision and proprioception, are updated (Burke et al., 1988). In contrast, when we perform an active movement, our brain generates and implements a motor plan that relies on internal simulations of our motor system (Wolpert et al., 1995): the motor plan is executed, monitored and corrected by comparing the efference copy with the generated sensory feedback (Miall and Wolpert, 1996; Todorov and Jordan, 2002). The implication of internal body simulations renders the processing of visuomotor cues during active movement much richer in body-related information content. Indeed, this is supported by experimental evidence. For example, it was shown that participants are better at discriminating synchronous vs. asynchronous visuomotor cues when performing active movements compared to passive (Shimada et al., 2010).

It is also relevant that visual monitoring and recognition of one's own actions has been proposed to contribute critically to the sense of body ownership (Jeannerod, 2003), and to play a major role in self-other discrimination (Jeannerod, 2004). Developmental studies have shown that, at 4- to 5-months of age, infants are already able to distinguish their limbs from those of other babies on the basis of synchronous visuomotor correlations: they can distinguish an online video stream of their own moving limbs from similar videos of other babies, from delayed videos of their own moving limbs, (Bahrick and Watson, 1985; Schmuckler, 1996), as well as from online videos in which their body spatial configuration is seen modified (Rochat and Morgan, 1995; Schmuckler, 1996).

A set of experimental studies has shown how healthy adults rely on visuomotor stimuli to discriminate whether a seen moving hand is their own. For example, in the experiment of Daprati and colleagues, participants were asked to perform a movement while looking at a screen in which their filmed hand or the experimenter's was displayed in the same position of their occluded hand. When asked to indicate whether the seen hand was their own hand, participants' performance showed a high rate of erroneous self-attribution when seeing the experimenter's hand doing the same movement (Daprati et al., 1997). In a similar setup, participants saw a virtual hand moving as their own occluded hand, under different spatial orientations and temporal delays. When asked to decide whether the displayed movement corresponded to their own movement, subjects made significantly more self-recognition errors for temporal delays smaller than 150 ms and for angular deviations smaller than 15° (Franck et al., 2001). A variation of the same setup was used to investigate how action and visual cues about the body spatial configuration are used in body-recognition processes (van den Bos and Jeannerod, 2002). Participants were presented with an online video of their own hand and the hand of the experimenter performing either the same, different or no movement at all, while displayed simultaneously in different orientations. The results showed that when the hands performed different movements, participants were almost always able to recognize their own hand. In contrast, when the visuomotor information was ambiguous (same movements) participants were less accurate and their responses were affected by the seen spatial configuration of the hands.

Overall, it becomes apparent that humans exploit the tight coupling of visual and motor signals not only for optimizing their movement performance, but also for attributing their body parts and body movements to themselves.

#### Methods and apparatus for visuomotor stimulation in BOIs

The induction of BOIs has been demonstrated in presence of visuomotor stimulation instead of visuotactile. In those experimental setups, the participants perform either active or passive movements while seeing the artificial body (or body part) moving (Figures 2B,E).

The animation of the fake body has been typically achieved through mechanical devices that permitted linking the real and the fake body parts, such as wooden rods (Kalckert and Ehrsson, 2012, 2014a), pulleys and strings (Azañón and Soto-Faraco, 2007), couplings (Walsh et al., 2011), pneumatically driven plungers (Riemer et al., 2013, 2014), and braces (Dummer et al., 2009). Alternative experimental setups used cameras to record the participants' moving hand and project it to a surface or a screen (Tsakiris et al., 2006, 2010b). The intrinsic delays of the apparatus are typically of the order of 70–100 ms (Tsakiris et al., 2006; Riemer et al., 2013, 2014), and thus below the threshold of 150 ms for detecting visuomotor delays (Franck et al., 2001; Shimada et al., 2010). Other experimenters, using virtual reality techniques, captured the participants' movements—through inertial systems and/or infrared cameras—and animated the virtual limbs or bodies accordingly (Sanchez-Vives et al., 2010; Yuan and Steed, 2010; Normand et al., 2011; Kilteni et al., 2012, 2013).

#### Spatial and temporal principles of visuomotor integration as trigger in BOIs

Several studies have demonstrated that BOIs are induced when both real and fake bodies move homologous body parts at the same time. This has been shown, for example, with fingers (Tsakiris et al., 2006, 2010b; Sanchez-Vives et al., 2010; Walsh et al., 2011; Kalckert and Ehrsson, 2012, 2014a,b; Riemer et al., 2013), hands (Dummer et al., 2009), arms (Yuan and Steed, 2010; Normand et al., 2011; Kilteni et al., 2012; Llobera et al., 2013), upper body (Kilteni et al., 2013), legs (Kokkinara and Slater, 2014), or full bodies (Banakou et al., 2013; Peck et al., 2013). In contrast, when introducing temporal delays of the order of 500 ms or more (Kalckert and Ehrsson, 2012, 2014a; Riemer et al., 2013, 2014), or when manipulating the seen movements independently from the real movement (Dummer et al., 2009; Sanchez-Vives et al., 2010; Walsh et al., 2011; Banakou et al., 2013), the corresponding illusions were inhibited. In addition to the temporal coupling, moving homologous real and fake body parts was also found to be essential for the induction of the illusion; for example, moving the index finger while seeing the artificial middle finger moving synchronously did not elicit the ownership illusion (Riemer et al., 2014).

#### Summary

Similarly to visuotactile stimuli, the experimental results suggest that the induction of BOIs depends critically on the spatiotemporal congruence between the seen and the felt movements.

### Visuoproprioceptive modulations and triggers for body ownership illusions

#### Visuoproprioceptive integration in own-body perception

Our own-body perception is greatly influenced by visual and proprioceptive information. The main contribution of visuoproprioceptive integration relies on defining where our body is in space. Both modalities inform the brain about where a specific body part is located at a given point in time. For example, we can estimate where our hand is with our eyes closed, and our judgement can further be confirmed by looking at our own hand.

Experimental evidence has shown that when vision and proprioception provide conflicting information, the perceived position of our body parts can significantly deviate from the veridical one. For example, when the hand is seen in a non-veridical location through mirrors or prisms, subjects perceive their hand to be in one single location, somewhere between the seen and the felt position (Hay et al., 1965; Pick et al., 1969; Warren, 1980) and closer to the visual one (van Beers et al., 1999). This visual capture of proprioception has been shown to influence the performance of reaching movements (Rossetti et al., 1995; Sober and Sabes, 2003; Holmes et al., 2004) and to play a crucial role in monitoring online movement execution (Lajoie et al., 1992; Sober and Sabes, 2003, 2005; Bagesteiro et al., 2006; Boulinguez and Rouhana, 2008). Interestingly, a recent study with 5–7 years old children has shown that the visual capture effects increase with age, suggesting a progressive maturation of visuoproprioceptive integration (Bremner et al., 2013).

Analogously to the visual capture of proprioception, it has been shown that in absence of direct vision of the body, proprioception can distort the perception of visual cues associated to the body spatial configuration. For example, participants sitting in a dark room while holding a luminous target with their static hand, report seeing the (static) luminous target moving in space when experiencing the kinaesthetic illusion of moving their (static) arm (Lackner and Levine, 1978). Similarly, moving the hand while seeing its afterimage (Gregory et al., 1959) was shown to distort the hand image or even to fade it out, despite that this should be perceptually static on the sole basis of visual input (Davies, 1973).

Most studies on visuoproprioceptive populations have been carried out in the context of reaching actions and motor control in both monkeys (e.g., Battaglia-mayer et al., 2000) and humans (e.g., Filimon et al., 2009). Nevertheless, neural populations have been found in the monkey brain, which integrate static visual and proprioceptive cues to encode the position of the arm (Graziano et al., 2000). To our knowledge, no analogous studies have been performed with humans.

Overall, experimental evidence suggests that visual and proprioceptive information significantly contribute to the perception of our own body and more particularly, in determining its perceived spatial configuration.

#### Methods and apparatus for visuoproprioceptive manipulations in BOIs

BOIs, including their full body versions, have been tested against differences between the seen spatial configuration of the fake body (or body part) and the one of the real body (or body part) that is experienced through proprioception.

In its original version (Botvinick and Cohen, 1998), the RHI was induced despite the spatial mismatch between the positions of the real and the rubber hand. The same spatial arrangement, with the rubber hand closer to the participants' body midline (Figure 2A), was quickly established as a standard protocol for subsequent studies (e.g., Armel and Ramachandran, 2003; Tsakiris and Haggard, 2005; Haans et al., 2008; Moseley et al., 2008; Schütz-Bosbach et al., 2009). In other studies, the rubber hand was instead placed above the occluded real one (e.g., Pavani et al., 2000; Ehrsson et al., 2004; Azañón and Soto-Faraco, 2007; Haggard and Jundi, 2009).

The above-mentioned studies used a physical object (i.e., the rubber hand) and their setups therefore entail intrinsic limitations in the possible manipulations of visuoproprioceptive stimuli; it is in fact never possible to have the two hands overlapping in space without using devices. Different techniques including mirrors (e.g., Longo et al., 2008a; Zopf et al., 2010), cameras (e.g., Petkova and Ehrsson, 2008; Hohwy and Paton, 2010) and virtual reality techniques (e.g., Perez-Marcos et al., 2012), permitted experimenters to overcome this limitation by projecting the fake body (body part) in complete overlap with its real counterparts.

Manipulations of the position and orientation of the fake body (body parts) relatively to the real body, allowed experimenters to investigate the extent to which visuoproprioceptive discrepancies affect the induction and strength of BOIs. Furthermore, it was possible to test whether close correspondence in the seen and felt spatial configurations (i.e., apparent spatial coincidence) can trigger BOIs without further crossmodal stimulation (e.g., visuotactile) (Figures 2C,F).

#### Visuoproprioceptive modulations of BOIs

##### The case of visuotactile triggers

As long as the fake hand was placed close to the body midline and thus within the reaching space, the RHI was induced in presence of spatiotemporally congruent visuotactile stimulation, for several tested mismatches between the positions of the real and fake hands in either the horizontal or the vertical plane; e.g., of 10–20 cm (e.g., Zopf et al., 2010; Rohde et al., 2011; Kalckert and Ehrsson, 2014b), 20–40 cm (e.g., Ijsselsteijn et al., 2006; Haans et al., 2008; Kalckert and Ehrsson, 2014b), or above 40 cm (e.g., Ehrsson et al., 2004; Zopf et al., 2010).

With respect to the horizontal distance, no significant differences were found in the strength of the RHI under small (i.e., 15 cm) or large (i.e., 45 cm) distances between the hands (Zopf et al., 2010), suggesting that the between hands' distance is not a crucial factor. Different results have been found when the rubber hand was placed farer away from the participant's body midline than the real one. In this case, increasing the horizontal distance between the two hands can eventually prevent the RHI even when the rubber hand still lies within the reachable space (Preston, 2013). This different effect of visuoproprioceptive mismatch on the elicitation of the RHI can probably be explained by our higher expectation of seeing our hand closer to the midline than away from it. The between hands horizontal distance was instead found to significantly affect subjective scores for conditions of asynchronous visuotactile stroking (Zopf et al., 2010): ownership scores were significantly higher when the two hands were as close as 15 cm with respect to the case in which the distance was larger (45 cm). In her study Preston (2013) did not find differences in questionnaire scores, which were equally low, independently on the between hands distance. The different results may be due to the different delays adopted in the asynchronous condition (about 300 ms and 1 s, respectively): in fact the delay used by Zopf and colleagues was reported to mark the upper limit for temporal discrepancies, above which the RHI is significantly weakened (Shimada et al., 2009, 2014).

With respect to the vertical plane, no significant differences in ownership scores were detected between placing the real and the fake hands at a vertical distance of 12 or 27.5 cm, though keeping both at the same horizontal position close to the participants' midline. However, increasing the vertical distance between the hands to 43 cm was found to significantly attenuate the RHI scores (Kalckert and Ehrsson, 2014b).

While in all the above-mentioned studies the real and fake hands differed in position but were mostly aligned in terms of orientation, other experiments have shown that BOIs can be induced in presence of an additional mismatch in orientation, provided that the fake body (body part) was seen in an anatomically plausible posture. For example, rotating the fake left hand by 44° clockwise did not prevent participants from experiencing the physical touch as if coming from the position of the rubber one, and further led to a recalibration of their perceived elbow joint angle (Butz et al., 2014). BOIs were experienced also when the seen fake hand, rotated away from the real one, crossed the body midline (Brozzoli et al., 2012; Perez-Marcos et al., 2012); but see (Costantini and Haggard, 2007; Cadieux et al., 2011) for effects in proprioceptive drifts. In addition, the RHI was induced toward a rubber hand placed palm-up when the fake palm was stroked synchronously with the palm of the real that was placed palm-down (Ionta et al., 2013). Moreover, by exploring different orientation mismatches, a recent study reported that tactile sensations were perceived to arise from the rubber mainly for those rotations that are easy to mimic with the real. Interestingly, the illusion onsets were found to be shorter when there were no orientation mismatches, without though any significant differences in the subjective reports (Ide, 2013). Instead, when discrepancies in both position and orientation were introduced by moving the rubber hand away from the participants' midline and rotating it, illusory tactile sensations were reported to gradually decrease in intensity with effects also in illusion onsets (Lloyd, 2007).

Consistent with these are the findings from full BOIs. These have been typically induced toward artificial bodies that appeared as if spatially coincident with the real ones, seen therefore from a 1 PP, and under spatiotemporally congruent visuotactile stimulation (Petkova and Ehrsson, 2008; Slater et al., 2010; Petkova et al., 2011b; Maselli and Slater, 2013). With respect to position, a recent study showed that a full BOI could be experienced toward a virtual body seen from a laterally shifted visual perspective (by about 25 cm), i.e., with the virtual body overlapping only partially with the real one, upon congruent visuotactile stimulation. When compared to a condition when the fake body was in complete overlap with the real one, no significant differences in ownership were detected (Maselli and Slater, 2014). Analogously for orientation, the illusion was induced when the mannequin's body was seen from a 1 PP but tilted upwards by 30° approximately (Petkova et al., 2011a).

##### The case of visuomotor triggers

Beside the information about the movement timings and the involved body parts, visuomotor stimuli provide information on how the relative position and orientation of the real and fake bodies change in time. Some of the studies that employed visuomotor stimulation of artificial body parts followed the RHI protocol and presented the artificial limb close to the midline and lateral to the real one, e.g., at a distance of 15–20 cm (e.g., Sanchez-Vives et al., 2010; Riemer et al., 2013, 2014) while others placed the fake limb above the real one at a vertical distance of about 12 cm (e.g., Walsh et al., 2011; Kalckert and Ehrsson, 2012, 2014a,b). With respect to the horizontal distance, no significant differences were found in the subjective reports of body ownership when the fake and the real limb moved synchronously while keeping a distance of about 10 cm, compared to when these were spatially coincident (Yuan and Steed, 2010). Nevertheless, larger distances were not tested. With respect to the vertical distance, a recent study revealed that small distances (i.e., 12 cm) permitted the induction of a robust RHI through congruent visuomotor stimulation while larger distances (i.e., 27.5 or 43 cm) significantly attenuated the subjective scores (Kalckert and Ehrsson, 2014b).

#### Visuoproprioceptive integration as trigger for BOIs

The fact that full BOIs were found to occur when having a static view of a highly realistic spatially coincident virtual body and under asynchronous visuotactile stimulation, suggests that congruent visuoproprioceptive cues alone could be sufficient to induce the illusion and further to sustain it under visuotactile discrepancies (Maselli and Slater, 2013). In partial agreement are findings from the RHI where participants seeing the rubber hand through a mirror, as if spatially coincident with their real one, did not disagree with statements of ownership when the visuotactile stimulation was asynchronous (Longo et al., 2008a).

In contrast to the large number of studies using visuotactile triggers, there have been fewer that explored whether spatial congruency of visuoproprioceptive cues can be by itself sufficient for the induction of BOIs. These studies, in which BOIs were assessed in “vision only” conditions, suggest that when there is neither visuotactile nor visuomotor stimulation the relative position and alignment of the real and fake body (body part) matters. When seeing through a mirror a rubber hand in the same position and orientation as the real one, participants gave positive scores for the illusion of ownership (Longo et al., 2008a; Giummarra et al., 2010). Nevertheless, upon direct comparison, additional synchronous visuotactile stroking elicited a significantly stronger illusion (Longo et al., 2008a). In contrast, when the fake and real hands were not spatially coincident, the mere vision of the rubber hand was not sufficient for inducing the illusion (as from participants' self-reports) (Rohde et al., 2011). In line with this overall evidence, it was shown that the visual exposure to a realistic spatially coincident virtual body by itself, can induce a full BOI (as reported in post experiment debriefing) (Maselli and Slater, 2014).

#### Summary

These findings reveal that spatial congruency of visuoproprioceptive cues (i.e., spatial coincidence) is not necessary for the illusion of body ownership to emerge, provided that the fake body (body part) is seen in an anatomical plausible configuration and in presence of congruent visuotactile or visuomotor stimulation. However, different degrees of visuoproprioceptive spatial mismatch significantly modulate both the intensity and the time onset of the illusion.

On the other hand, spatial coincidence may be a sufficient condition for eliciting BOIs. Although there is consensus in the literature that congruent visuotactile and visuomotor stimulation is necessary for inducing BOIs, this may specifically apply to conditions that include visuoproprioceptive mismatches.

### Semantic constraints in body ownership illusions

#### Semantic constraints in own-body perception

Beside the continuously updated sensory and motor information, our own-body perception largely relies upon higher-order, cognitive processes. Our semantic memories and knowledge contribute in shaping an abstract body model that contains information about the general and not self-specific visual, postural and structural properties of the human body. For example, we know how the human body is structured (e.g., the body has two hands), as well as how many degrees of freedom it has when it moves.

Given their non-self-specific nature, these cognitive processes depend, to an important extent, on neural mechanisms and brain areas that evolved for the visual perception of others' human bodies. Apart from brain areas specifically devoted to face perception (Haxby et al., 2000), several specialized regions for visual processing of bodies have been found in humans and non-human primates. This applies to hands, bodies (with and without heads), and to anatomically plausible body postures and motion (Peelen and Downing, 2007). Research in infants has shown that these selective areas are already functional at few days/months (Gliga and Dehaene-Lambertz, 2005; Hirai and Hiraki, 2005; Reid et al., 2006; Simion et al., 2008), suggesting that we are born equipped with structures for the visual encoding of body parts and human body kinematics.

Moving the focus away from own-body perception for a moment, semantic information has been proposed to be an important feature of multisensory integration. When considering two or more crossmodal stimuli, semantic congruence refers to their “close correspondence of content” (Doehrmann and Naumer, 2008). For example, a visual stimulus showing a dog is semantically congruent with a barking but not with a meowing sound (Alais et al., 2010). In this case, the semantic congruence of the audiovisual stimuli speaks in favor of a common underlying cause: a dog. Several studies have shown that the integration of crossmodal stimuli is enhanced when these are semantically congruent (Doehrmann and Naumer, 2008).

The relevance of semantic congruence in the context of own-body perception has been demonstrated in non-human primates with the seminal work of Graziano et al. (2000). The authors showed that the activity of visuoproprioceptive neurons in the monkey brain is importantly modulated by the semantic content of the visual stimulus. Single cell activity was recorded while manipulating the position of the occluded monkey's arm, and that of a seen object. The object was either a replica of the arm or another object, such as a white paper or an apple's slice. The activity of the neurons was significantly modulated by the position of the arm replica, if the latter was in an anatomically plausible configuration. In contrast, changing the position of the white paper, or of the apple slice, had no effect. Interestingly, if the arm replica was seen in non-anatomically plausible configurations, i.e., with opposite handedness or rotated by 180° so that the fake fingers were pointing to the chest, the observed activity was no longer affected by the fake arm's position (Graziano et al., 2000).

Altogether, experimental evidence suggests that our brain is equipped with highly specialized structures for the visual processing of body parts and, in order to be effectively integrated with the somatosensory signals, visual cues should not only resemble body parts but further satisfy body semantic constraints in terms of anatomical plausibility of posture and structure.

#### Semantic constraints in BOIs

**Figure 3:**
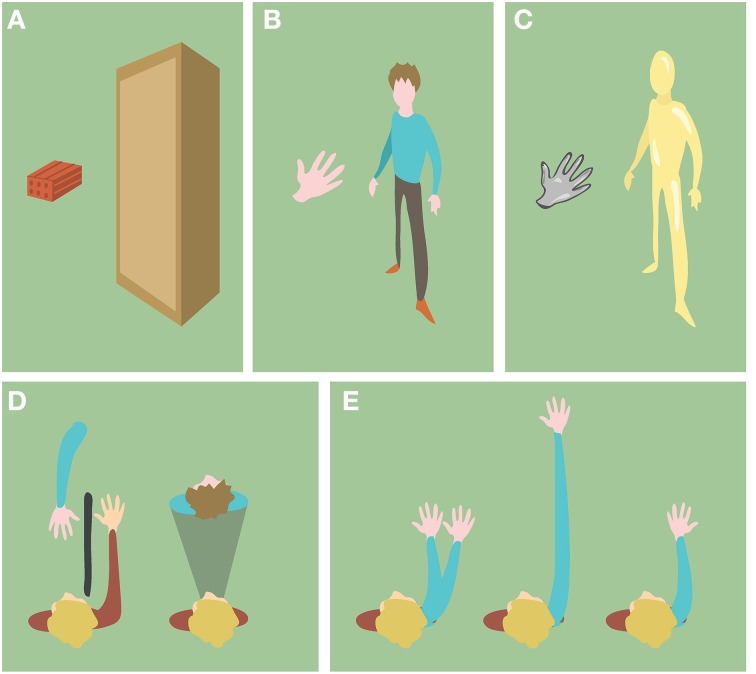
**Examples of objects with different semantic information. (A)** Objects with non-human body shape. **(B)** Objects with human body shape. **(C)** Objects with non-human skin texture **(D)** Objects (blue) in anatomically implausible spatial configurations with respect to the participant's body (red). **(E)** Objects (blue) with anatomically implausible structure with respect to the human body.

In the particular context of BOIs, the level of semantic congruence refers to which extent the non-corporeal object resembles a not self-specific human body (body part) in terms of shape, anatomy and structure. We first distinguish between objects with human body shape or not (Figures 3A,B). If the objects are body-shaped, their semantic information can be further characterized by their texture, the anatomical plausibility of their spatial configuration and the anatomical plausibility of their internal structure (Figures 3C–E).

#### The role of shape

Converging experimental evidence suggests that BOIs are shape-sensitive. For example, when a checkerboard (Zopf et al., 2010) was used instead of a rubber hand, the reports of the illusion were weaker if not entirely absent. Analogous inhibition in subjective reports was found for non-body shaped objects with hand-like skin texture (Haans et al., 2008) or size (Hohwy and Paton, 2010, experiment 3). Similar inhibitory effects were also detected for proprioceptive drifts when using a stick instead of a rubber hand (Tsakiris and Haggard, 2005). Analogously, full BOIs were suppressed when participants had a 1 PP view of a rectangular body-sized object instead of a 1 PP view of a mannequin (Petkova and Ehrsson, 2008). Furthermore, even smaller violations of shape were shown to have an effect; in the study of Tsakiris et al. (2010a) the RHI was reported only for the rubber hand and not for other objects, including a wooden block with sculpted wrist and fingers.

The results reported in the study of Armel and Ramachandran (2003) are somewhat in disagreement with these findings: stroking a table surface synchronously with the participants' hidden hand induced weak ownership sensations toward the table, though significantly higher in intensity compared to the control condition in which the real hand was visible. Despite this, subjective scores for the “table ownership” were significantly lower with respect to when a rubber hand was used–a condition that always preceded the table one. Motivated by this point, the study of Hohwy and Paton (2010) showed that while the mere synchronous visuotactile stimulation of a piece of hand-sized cardboard was not sufficient to induce the illusion (experiment 3), prior induction of the illusion (by using a rubber hand) could instead allow the illusion to be sustained and projected on the cardboard when the rubber hand was instantaneously replaced by it (experiment 2).

These results suggest that whereas BOIs are critically sensitive to shape, a prior exposure to the illusion can modulate this sensitivity by overriding the contribution of prior knowledge about the shape of human bodies to the illusion itself.

#### The role of texture realism

Semantic information related to the realism of the fake body (body part) has been investigated through modifying its seen material texture. The realism of the seen texture is associated with the degree of the perceived biological plausibility of the seen object as a candidate for a human body part. Texture realism has been shown not to be crucial for BOIs; yet, modulations in the intensity of the illusion have been found when manipulating the texture. For example, the RHI has been elicited toward a fake hand wearing a latex glove, though at a significantly lesser intensity compared to when the realistically textured rubber hand was used (Haans et al., 2008). Similarly, a mechanical hand-shaped object made of wires, permitted the induction of the RHI through congruent visuotactile stimulation, albeit to a significantly lesser extent compared to when a realistic replica of a human hand was used (Bertamini and O'Sullivan, 2014). Turning to full BOIs, texture realism was proposed to have a modulatory effect when proving visuotactile stimulation (Maselli and Slater, 2013): whilst congruent visuotactile triggers were found to be necessary in eliciting the full BOI toward a spatially coincident plastic mannequin (Petkova and Ehrsson, 2008; Petkova et al., 2011b), using a realistically textured virtual body produced negligible differences between congruent and incongruent visuotactile stimulations (Maselli and Slater, 2013). Nevertheless, congruent visuomotor stimulation with a spatially coincident virtual body having an unnatural purple skin color induced the illusion, without significant differences in strength compared to when a realistic skin texture was used (Peck et al., 2013). This last result suggests that visuomotor correlations may tend to saturate the illusion, so that modulations by texture become negligible.

#### The role of anatomical plausibility of spatial configuration and of visual perspective

The anatomical plausibility of the spatial configuration of the fake body was shown to play a critical role in the induction of BOIs. For example, rotating the rubber hand by 180° (with the rubber fingers pointing toward the participant) prevented the RHI, despite congruent visuotactile (Ehrsson et al., 2004; Holle et al., 2011) or visuomotor (Kalckert and Ehrsson, 2012) stimulation. Analogously, a 90° rotation of a left rubber hand (with the rubber fingers pointing to the left) was shown to reduce the difference in proprioceptive drifts between synchronous and asynchronous visuotactile stimulation (Tsakiris and Haggard, 2005) and to delay the onset of the RHI (Aimola Davies et al., 2013). A systematic study in which different rotations were applied on the rubber hand, including both anatomically plausible and implausible configurations, has shown that illusory experiences were elicited mainly for anatomically plausible configurations (Ide, 2013).

Apart from violating constraints of human joints articulation, the plausibility of the body spatial configuration can be broken also by manipulating the visual perspective on the fake body or body part. For example, seeing directly one's own body from a distance is anatomically impossible. Similar to findings from the RHI, having a 3 PP over a distant virtual body or a mannequin was found to inhibit the illusion, despite congruent visuotactile stimulation between the real and the fake body. Upon direct comparison of 1 PP and 3 PP conditions, this inhibition was consistently found for different distances at which the fake body was seen: 100 cm (Slater et al., 2010), 80 cm (Maselli and Slater, 2013), 75 cm (Petkova et al., 2011b), and 40 cm (Maselli and Slater, 2014). In contrast, when the visual perspective was slightly shifted from the eyes of the virtual body, but still consistent with an anatomically plausible view of the own body, the full BOI was induced (Maselli and Slater, 2014). These results are consistent with evidence from RHI experiments that have shown an inhibition of the illusion for rubber hands placed beyond the reaching space. These, as well as other configurations, e.g., the fake hand rotated 180°, could be regarded as cases of having a 3 PP over the fake limb. Interestingly, Bertamini and colleagues induced a vivid RHI providing exclusively a 3 PP view of the rubber hand through a mirror (Bertamini et al., 2011). The apparent disagreement between this result and the findings mentioned above could be explained by the fact that the 3 PP view was provided through a mirror, and considering that we automatically attribute visual information from the mirror to the physical location of the reflected objects. Indeed, the critical role of the mirror in mediating the visual perspective was confirmed within the same study: the RHI was abolished when providing exactly the same visual information about the rubber hand and visuotactile stimulation, but without an intervening mirror.

#### The role of anatomical plausibility of structure

The impact of anatomical plausibility of the structure of the seen object has been shown to vary importantly depending on the specific manipulation. For example, full BOIs have been shown toward artificial bodies of a significantly different scale from the real ones (van der Hoort et al., 2011; Banakou et al., 2013). With respect to the volume of the artificial body (body part), several studies have successfully elicited BOIs toward two dimensional video projections of either the real or the rubber hand (Ijsselsteijn et al., 2006; Tsakiris et al., 2006; Shimada et al., 2009) or toward a normal-sized hand of non-biological texture and of reduced volume (Bertamini and O'Sullivan, 2014), although the illusion was found to be stronger when the fake body part was seen with normal volumetric characteristics (Ijsselsteijn et al., 2006). Concerning body connectivity, subjective scores of ownership toward a virtual hand were significantly stronger when this was seen to be connected to the virtual torso rather than disconnected (Perez-Marcos et al., 2012).

With respect to body proportions, several studies that manipulated the seen hands to be either smaller or larger than the average population size, revealed various effects, including weight and size perception (e.g., Haggard and Jundi, 2009; Bruno and Bertamini, 2010; Linkenauger et al., 2013), distance perception (e.g., Linkenauger et al., in press), and movement kinematics (e.g., Bernardi et al., 2013). When studying the particular effect of hand size on eliciting the RHI, high illusion scores without significant differences were reported when using either a small (e.g., hand size of a primary school child) or a large (e.g., hand size of a tall man) rubber hand (Heed et al., 2011). Similarly, no significant differences in the illusion scores were detected when using a two dimensional hand image that was either equal in size, magnified or shrunk by 3 cm with respect to the participants' real hand (Pavani and Zampini, 2007). Similarly, the RHI was elicited with a small and a large rubber hand, still within the range of anatomically plausible body structures (Bruno and Bertamini, 2010). In contrast, using artificial body parts that violated normal body proportions, as for example a fake arm 91 cm beyond the real one, was shown to elicit weaker feelings of ownership through congruent visuotactile stimulation, compared to when the fake arm was seen with a plausible length (Armel and Ramachandran, 2003). When providing both visuotactile and visuomotor stimulation, participants gave high scores of ownership over a virtual arm up to three times the length of the real one, but less strongly at four times the length (Kilteni et al., 2012).

A similar effect was found when manipulating the number of artificial body parts. For example, stroking a rubber hand synchronously with the participants' visible hand (Guterstam et al., 2011), or stroking two rubber hands placed side-by-side synchronously with the participants' occluded real hand (Ehrsson, 2009), was shown to induce the illusion of having multiple limbs or owning two hands of the same laterality at the same time, respectively. Nevertheless, in both cases the experienced ownership was stronger when employing the classic RHI setup. With respect to simultaneous ownership toward two rubber hands of the same laterality, it was shown that these need to be placed symmetrically with respect to the real limb (Folegatti et al., 2012). High illusion scores concerning having two limbs were further reported when participants were moving their hand while seeing two video replicas, projected symmetrically with respect to the real, moving accordingly (Newport et al., 2010).

The spatial arrangement of the artificial body part with respect to the participant's body was instead demonstrated to be critical. For example, seeing a right rubber foot being stroked synchronously with the participants' right hand did not induce ownership feelings (Guterstam et al., 2011). Analogously, no significant differences were found in proprioceptive drifts when comparing synchronous and asynchronous stimulation applied on a right rubber hand and the participants' left hand (Tsakiris and Haggard, 2005).

#### The role of stimulation congruence

Another aspect of semantic congruence concerns the compatibility of the seen and felts touch when inducing BOIs through visuotactile stimulation. In the majority of studies, the instrument seen to administer the touch on the artificial body was the same—e.g., paintbrushes (Botvinick and Cohen, 1998), sharp pins (Capelari et al., 2009), rods (Petkova and Ehrsson, 2008)—or a visually compatible one, with the one used to deliver the touch on the real body (Slater et al., 2008; Pabon et al., 2010; Normand et al., 2011; Maselli and Slater, 2013). Nevertheless, using different instruments could inhibit the occurrence of the BOI by violating the participants' tactile expectancies: the participant who sees the fake body being touched by a soft texture material, would expect sensations of analogous quality on his/her real body. This was investigated by using a piece of cotton to stimulate the rubber hand while using a piece of sponge to stimulate the real hand or vice versa. Results from both proprioceptive drifts and questionnaires were not significantly different from the case in which the same instrument was used (Schütz-Bosbach et al., 2009).

#### Summary

Experimental evidence suggests that BOIs are greatly affected by the semantic information the visual cues of the fake body bring along, at least in terms of their shape, the anatomical plausibility of their spatial configuration and structure. Even if some forms of anatomical violations (e.g., larger or smaller bodies, longer or multiple limbs) can be tolerated and overcome by congruent multimodal stimulation, other forms of violation (e.g., spatial arrangement) may not. This suggests that, in order to be perceived as parts of the own body, fake objects need to satisfy to some extent semantic constraints from an abstract and not self-specific body model (Tsakiris et al., 2007; Longo et al., 2009; Tsakiris, 2010). Yet, the exact boundaries of tolerable violations are still to be determined.

## Theoretical accounts for induction of body ownership illusions

The studies on BOIs that we have reviewed so far provide important insights on the contribution of multisensory signals and semantic knowledge on the emergence of our sense of body ownership. However, in order to gain a deeper understanding on how the brain infers which is our body and its limbs, it is necessary to understand why BOIs are induced at all. Although there is extensive consensus in the literature that multisensory processing of bodily signals underlies the induction of BOIs (Graziano and Botvinick, 2002; Makin et al., 2008; Tsakiris, 2010; Ehrsson, 2011; Maselli and Slater, 2013), very few researchers attempted to cast the experimental conclusions into a unitary framework.

The initial Botvinick and Cohen's proposal was that the RHI arises from the three-way interaction between vision, touch and proprioception (Botvinick and Cohen, 1998). This idea was reflected in the proposal of Makin et al. (2008). According to their model, visual and proprioceptive cues about the positions of the real and rubber hands are first combined into a single estimate of the hand's spatial configuration; following, visual and tactile information about the seen and felt touches are processed in the common reference frame of the hand and, if integrated, elicit the illusion. This proposal was extended to account for experimental findings on the contribution of semantic information. According to the model proposed by Tsakiris (2010), the integration of multisensory cues is gated by semantic information in terms of the shape and anatomical plausibility of the seen object; only those objects that fit a pre-existing internal model of the human body can be therefore experienced as parts of the own body. In his account, Tsakiris proposed that this “test-for-fit” process takes place in the right temporo-parietal junction (Tsakiris et al., 2008). Alternatively, it was suggested that this selection process might be operated by visuoproprioceptive populations (Maselli and Slater, 2013).

Although these models offer a schematic description of the induction of BOIs, by referring also to candidate brain areas that operate the multisensory integration and the semantic gating, they do not explain what is the underlying computational problem, and how our brain solves it with the resulting illusory experience. In the following, we briefly review recent predictive coding accounts that propose a specific computational framework for the study of self-processing, including the sense of body ownership. Subsequently, we focus on two alternative computational accounts, namely the Bayesian causal inference models and the connectionist models. Both approaches have been developed to explain various multisensory perceptual phenomena and they could be adopted and extended in future works to address the problem of BOIs.

### Predictive coding approaches

In a recent stream of work (Hohwy, 2007; Apps and Tsakiris, 2013; Clark, 2013; Limanowski and Blankenburg, 2013; Seth, 2013), the problem underpinning self-processing and self-recognition has been approached within the general framework of hierarchical generative models, such as predictive coding (Srinivasan et al., 1982; Rao and Ballard, 1999) and the free-energy principle (e.g., Friston, 2009). These models adopt the view, originally put forward by Von Helmholtz (Hatfield, 1990), that the brain needs to infer the hidden causes of the sensory signals; for example, the existence of a dog when hearing barking-like sound. It does so, by minimizing the error between the predictions it makes about the expected cues based on its internal causal model (e.g., what would be the auditory stimulus associated with a dog) and the available sensory information (e.g., the heard barking sound).

In this context it has been proposed that the RHI results from minimizing the prediction errors that arise from feeling the touch on the real hand and seeing the touch at a different location (i.e., on the rubber hand), as well as the errors concerning the rubber hand not looking exactly as the real hand. Error minimization would thus result in merging the spatial representation of the two hands and further in updating prior beliefs about one's own hand appearance (Apps and Tsakiris, 2013; Seth, 2013). Interestingly, several findings from RHI studies can be explained as the effect of strategies for error minimization; this is the case of the attenuation of somatosensory processing in the real hand, like e.g., the slowing of tactile temporal processing (Moseley et al., 2008), the increase of tactile detection threshold (Zopf et al., 2011), and the reduction of primary somatosensory cortex responses to tactile stimuli (Zeller et al., 2014). Since the main source of errors arises from the spatial discrepancies between the visual position of the rubber hand and the position of the real one provided by somatosensation, decreasing the precision of the latter could constitute an effective strategy to minimize the error (Zeller et al., 2014).

Despite the insights that these models provide about possible neurobiological mechanisms underlying BOIs, to date these proposals have been formulated mainly at a conceptual level, without tackling the underlying computational problem.

### Causal inference in multimodal cue integration under BOIs

In contrast to predictive coding accounts, Bayesian causal inference models offer a simpler and comprehensive computational account for multisensory phenomena, usually without addressing the neurobiological mechanisms. According to these models, when people are presented with two stimuli from different modalities, they initially infer whether these have the same origin (i.e., cause) or not, and then they combine their information according to these beliefs (Körding et al., 2007). For example, when we hear a barking sound and we see a dog close to the auditory source, we will be confident that the barking sound comes from the seen dog and we will therefore use both the visual and the auditory information to estimate the position of the dog. In contrast, when the sound is perceived to come from a very distinct position with respect to the position of the seen dog, we may consider the existence of two dogs as more likely. Causal inference models were shown to explain various phenomena in multisensory perception (Shams and Beierholm, 2010), including the spatial (Körding et al., 2007; Wozny et al., 2010) and temporal (Shams et al., 2005) ventriloquist effects, and visuomotor adaptation (Körding and Tenenbaum, 2006; Wei and Körding, 2009).

While these models have been typically used to describe the combination of two sensory cues, analogous ideas could be applied to explain the induction of BOIs. Indeed, Armel and Ramachandran (2003) proposed that the RHI is induced due to a Bayesian perceptual learning driven by the high likelihood of the visual and tactile stimuli to occur from one common event. In other words, it is more likely that the seen touch and the felt touch belong to the same event (i.e., my hand being stroked), than the existence of one artificial hand that is stroked synchronously with my hidden hand. Due to the fact that their proposal was exclusively based on the likelihood of sensory data, it was quickly put aside by other authors, as it was unable to explain the inhibition of the illusion under semantic violations (Tsakiris and Haggard, 2005; Tsakiris, 2010).

**Figure 4:**
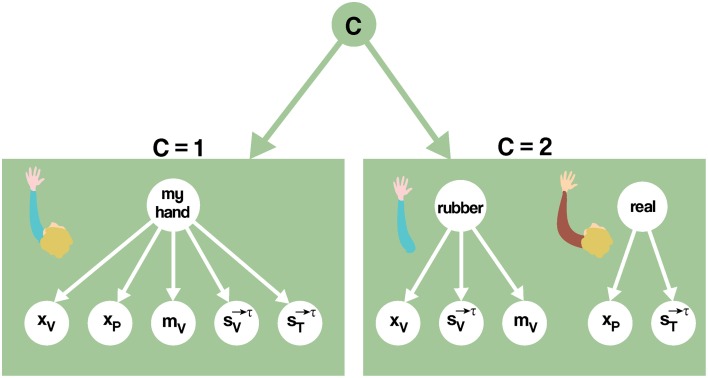
**A causal inference model for the classic version of the RHI**. Left: one cause being responsible for all cues. In this case, the visually perceived configuration x_V_, the proprioceptive perceived configuration *x*_P_, the seen strokes ${\text{s}}_{\text{V}}^{\to \tau }$ and the felt strokes ${\text{s}}_{\text{T}}^{\to \tau }$ together with the seen morphological characteristics *m*_V_, are mapped into a common cause (*C* = 1). Right: alternatively, two distinct causes may be inferred, decoupling the problem into two independent estimation problems. The brain infers whether the seen and felt spatial configurations, the tactile events and the seen morphology origin from the same causal structure, i.e., my hand (*C* = 1), or independent causal structures, i.e., the real and the rubber hands (*C* = 2), and then derives optimal predictions from this.

Nevertheless, a Bayesian model that includes semantic influences can be used to address the induction of RHI. For example, using the causal inference framework, the computational problem the brain needs to solve refers to inferring whether there is a common cause (i.e., my hand) or two different causes (i.e., my hand and the rubber one) generating the available visual, tactile, and proprioceptive signals (Figure 4). Mathematically formalizing the problem, the nervous system needs to calculate the probability of there being one hand (C = 1) vs. there being two hands (C = 2), given the available sensory information and prior knowledge. At time τ, the sensory data consist of the static visual (x_V_) and proprioceptive (x_P_) spatial configuration of the real and rubber hands, the semantic information of the seen object m_v_, and the trains of tactile stimuli applied, up to the current moment τ, on the rubber (${\text{s}}_{\text{V}}^{\to \tau }$) and the real (${\text{s}}_{\text{T}}^{\to \tau }$), respectively (**Equation 1**).

(1) $$\begin{array}{l} \text{p}(\text{C}=1|{\text{m}}_{\text{V}},{\text{x}}_{\text{V}},{\text{x}}_{\text{P}},{\text{s}}_{\text{V}}^{\to \tau },{\text{s}}_{\text{T}}^{\to \tau })\;\\ \;=\frac{\text{p}({\text{m}}_{\text{V}},{\text{x}}_{\text{V}},{\text{x}}_{\text{P}},{\text{s}}_{\text{V}}^{\to \tau },{\text{s}}_{\text{T}}^{\to \tau }|\text{C }=\;1)\text{p}(\text{C }=\;1)}{\text{p}({\text{m}}_{\text{V}},{\text{x}}_{\text{V}},{\text{x}}_{\text{P}},{\text{s}}_{\text{V}}^{\to \tau },{\text{s}}_{\text{T}}^{\to \tau })} \end{array}$$

Under the simplifying assumption that the visual, tactile and proprioceptive information are conditionally independent, the problem can then be formulized as:

(2) $$\begin{array}{l} \text{p}\left(\text{C}=1|{\text{m}}_{\text{V}},{\text{x}}_{\text{V}},{\text{x}}_{\text{P}},{\text{s}}_{\text{V}}^{\to \tau },{\text{s}}_{\text{T}}^{\to \tau }\right)\\ \;=\frac{\text{p}\left({\text{m}}_{\text{V}}|\text{C}=1)\text{p}({\text{x}}_{\text{V}},{\text{x}}_{\text{P}}|\text{C}=1)\text{p}({\text{s}}_{\text{V}}^{\to \tau },{\text{s}}_{\text{T}}^{\to \tau }|\text{C}=1\right)\text{p}\left(\text{C}=1\right)}{\text{p}\left({\text{m}}_{\text{v}},{\text{x}}_{\text{V}},{\text{x}}_{\text{P}},{\text{s}}_{\text{V}}^{\to \tau },{\text{s}}_{\text{T}}^{\to \tau }\right)} \end{array}$$

The model described by Equations (1) and (2) states that the probability for a single hand existing, estimated at time τ given the sensory evidence, depends on the likelihood of the evidence given only one hand—p(m_V_, x_V_, x_P_, ${\text{s}}_{\text{V}}^{\to \tau }$, ${\text{s}}_{\text{T}}^{\to \tau }$ | C = 1)—and on the prior expectation for only one hand being there —p (C = 1). The likelihood of the data depends on (i) the distance between the two hands, i.e., p(x_V_, x_P_ | C = 1), (ii) the spatiotemporal discrepancies of the visuotactile stimulation, i.e., p(${\text{s}}_{\text{V}}^{\to \tau }$, ${\text{s}}_{\text{T}}^{\to \tau }$ | C = 1), and (iii) the level of semantic congruence between the seen hand and the real one, i.e., p(m_V_ | C = 1). These visuoproprioceptive, visuotactile, and semantic factors contribute to the likelihood by increasing it in proportion to the congruency of the correspondent stimuli^1^. The likelihood, in turn, is in continuous interplay with prior expectations (e.g., the position of the limbs close to the body midline, personality traits including suggestibility to illusory perceptions, previous experience of the illusion), to update the posterior probability of only one hand being responsible for the sensory inputs, p(C = 1 | m_V_, x_V_, x_P_, ${\text{s}}_{\text{V}}^{\to \tau }$, ${\text{s}}_{\text{T}}^{\to \tau }$). The illusion is then experienced when this posterior probability exceeds a given threshold (Wozny et al., 2010).

This relatively simple model, at least qualitatively, can accommodate many of the experimental findings on the RHI. For example, when the rubber hand is placed close to the real hand and without violating anatomical constraints, spatiotemporally congruent visuotactile stimulation will accumulate evidence in favor of a common hand being the source of all sensations and would induce the illusion (e.g., Botvinick and Cohen, 1998). The progressive convergence of the model to a common hand scenario is also supported by experimental evidence that the RHI is not immediate but needs time to be elicited (Peled et al., 2000; Ehrsson et al., 2004; Perez-Marcos et al., 2012). On the other hand, when the visuotactile stimulation is asynchronous (e.g., Botvinick and Cohen, 1998; Armel and Ramachandran, 2003) or spatially incongruent (e.g., Kammers et al., 2009), the evidence will favor the existence of two hands, and the illusion will not be induced. In addition, semantic violations in terms of shape, anatomical plausibility and realism will render the contribution of the likelihood less important and therefore, the induction of the illusion more difficult, and its resulting strength weaker (Armel and Ramachandran, 2003; Haans et al., 2008; Ehrsson, 2009; Ide, 2013). Moreover, placing the rubber hand far from the real one and from the participant's midline corresponds to decreasing both the likelihood of visuoproprioceptive signals and the prior probability of the limb's position (Lloyd, 2007; Preston, 2013). Finally, when the two hands are overlapping or close in space, the high likelihood of visuoproprioceptive information can render the contribution of the visuotactile likelihood unnecessary (Giummarra et al., 2010) or less relevant (Zopf et al., 2010).

This formulation of the problem could be further extended to dynamically include the effects of having experienced the illusion at a given time, on the (prior) expectation for the illusion to occur at following times, as well as on the way the brain processes the incoming sensory signals (likelihood) later on. Computationally this could be done by adopting an iterative approach in which both the prior expectation and the probability densities representing the likelihoods at a given time step, evolve over time as a function of the posterior probability. Such an extension of the model could account for several aftereffects reported in the literature. In Hohwy and Paton (2010), for example, it was reported that if, after a period of congruent visuotactile stimulation in which the illusion is established, the stimulation switches to incongruent patterns (e.g., seeing elevated stroking on the rubber hand while receiving physical touch) then participants would keep experiencing the illusion together with unusual perceptual sensations (e.g., supernaturally caused tactile sensations). These results can indeed be explained by the fact that having experienced the illusion enhances the expectation for the illusion to be there (increasing the prior probability) and, at the same time, this modulates the probability densities of the likelihoods involved (p(${\text{s}}_{\text{V}}^{\to \tau }$, ${\text{s}}_{\text{T}}^{\to \tau }$ | C = 1) in this case), e.g., increasing the variance, so to “explain away” the ongoing illusion and suppress the sensory conflict. This formulation is conceptually analogous to minimizing prediction errors in predictive coding approaches and, as discussed in the previous section, can account for several after-effects of the illusion, e.g., the attenuation of the somatosensory processing on the real hand (see the previous section for references).

It is noteworthy that this type of model can be adapted to include the effects of patterns of visuotactile stimulations that go beyond spatiotemporal congruency, such as applied pressure, duration, trajectory, frequency, velocity or predictability. Recent studies have observed that some of these features, closely related to affective and emotional aspects of touch, (e.g., the velocity of the applied stimulation) can significantly modulate the RHI (Crucianelli et al., 2013; Lloyd et al., 2013; van Stralen et al., 2014). Particularly concerning the predictability of the visuotactile stimulation, the probability of two independent visuotactile patterns being spatiotemporally congruent is lower the higher their complexity. Therefore, being exposed to congruent patterns of increasing complexity should lead to stronger illusions, and/or to illusions established on shorter time scales. Although there is no study explicitly testing this, reports in the literature support that increasing the stimulation variability can avoid habituation effects and lead to a faster and stronger illusion (Niebauer et al., 2002; Armel and Ramachandran, 2003; Tsakiris et al., 2008; Petkova and Ehrsson, 2009; Guterstam et al., 2011). The same observations were reported for visuomotor stimulation (Tsakiris et al., 2006; Kalckert and Ehrsson, 2012). This evidence is consistent with the proposal that unpredictability increases the weight of multisensory likelihood in inferring a common body.

A similar causal inference model can be applied to address the induction of BOIs in presence of visuomotor stimuli. In this case, visual and proprioceptive inputs that change dynamically in time can provide accumulating evidence in favor of a single cause if congruent—p (C = 1 | m_V_, ${\text{x}}_{\text{V}}^{\to \tau }$, ${\text{x}}_{\text{P}}^{\to \tau }$). Such a formulation can further bring forward the question of whether some crossmodal stimuli contribute more in inferring our own body than others. Congruent visuotactile or visuomotor stimuli between the real and rubber index finger was shown to elicit the RHI without significant differences (Riemer et al., 2013; Kalckert and Ehrsson, 2014a). Nevertheless, when testing the relative role of visuomotor and visuotactile correlations between the real and the virtual lower limbs under the same experimental condition, a stronger contribution of visuomotor stimuli was found in inducing the BOI, whilst incongruent stimulation of each of the two led equally to its break (Kokkinara and Slater, 2014). Given that visuomotor correlations contain a larger amount of information compared to visuotactile correlations (e.g., in terms of the internal model involved), the proposed model would predict a faster onset of BOIs. Moreover, this facilitation might also depend on which body parts are involved in the movements (e.g., moving the index finger vs. moving an entire upper or lower limb). However, these predictions need to be explicitly tested.

Finally, the same model could be reformulated and extended to account for the full BOIs taking into account the relevant pieces of multimodal and semantic information.

### Network models

Connectionist models offer an alternative approach for addressing the problem of BOIs. Although they have not been previously envisioned in this context, these models have been successfully applied in the field of multisensory integration as an alternative approach to Bayesian models (e.g., Patton and Anastasio, 2003; Martin et al., 2009; Cuppini et al., 2011). Connectionist models implement artificial networks with architectures that are typically inspired from neurobiological structures and are suited to simulate learning mechanisms and collective neuronal behavior. They therefore offer an important advantage with respect to the Bayesian approaches, as they can provide deeper insights into the neural mechanisms involved in the processes under study, although at the expense of higher complexity (Fernandes and Kording, 2010)

Apart from reproducing some of the most relevant properties of multisensory processing at the level of neural activity (Patton and Anastasio, 2003; Rowland et al., 2007; Martin et al., 2009), recent implementations have been proposed to account for perceptual illusions. For example, relatively simple networks, consisting of two layers reciprocally interconnected, could reproduce common illusions such as the sound-induced flash illusion and fusion (Cuppini et al., 2014), as well as the ventriloquist effect and aftereffect (Magosso et al., 2012).

Particularly relevant for the present discussion is the implementation of neural networks whose dynamics reproduces several observed properties of visuotactile processing. For example, a network with two unimodal and one bimodal areas, could reproduce effects of visuotactile integration such as the facilitation of tactile detection, localization and acuity, by concurrent visual information (Magosso, 2010). Modifications of this implementation have been shown to reproduce other key aspects of visuotactile integration. For example, by including Hebbian learning mechanisms tuned by attentional effects (Ursino et al., 2007; Magosso et al., 2010b), it was possible to simulate the dynamical expansion of the peripersonal space observed after tool use (Maravita and Iriki, 2004). In addition, interhemispheric competition effects, similar to the ones observed in right-brain-damaged patients (di Pellegrino et al., 1997; Mattingley et al., 1997), have been further emulated with models in which two replications of the network described above (one for each hand) were interconnected by inhibitory interneuron modules (Magosso et al., 2010a,b).

All these remarkable results address the possibility to apply this class of model to develop new theoretical accounts for BOIs. A plausible scenario would be to initially combine these models with Bayesian modules. For example, while the visuotactile processing involved in the RHI could be simulated with neural networks, the test-for-fit gating associated with neural populations specialized in body parts recognition, could be more conveniently described with the Bayesian formalism. Alternatively, existing implementations of neural networks for the representation of semantic information (for a review see Ursino et al., 2014), provide a valid option for representing sematic factors in BOIs. Such envisioned hybrid models would be indeed ideal for gaining deeper insights into our current understanding of the BOIs and their implications for own-body perception.

## Conclusions

One of the important questions in neuroscience, psychology, and philosophy concerns how we distinguish our body from the bodies of others as well as from objects in the surrounding environment (Gallagher, 2000; Jeannerod, 2003; Blanke and Metzinger, 2009; de Vignemont, 2011). Body ownership illusions (BOIs) are a powerful experimental tool to address this question, since they permit to investigate the conditions under which we can perceive artificial body parts or fake bodies as belonging to ourselves. In the present review, we focused on the role of multisensory integration and semantic knowledge in inducing BOIs. In particular, we reviewed the role of the temporal, spatial, and semantic relationship of crossmodal stimuli in the elicitation of the illusions as reflected in several experimental studies, and we further discussed and proposed theoretical accounts in order to cast all this information into one computational framework.

Given the vastness of the experimental literature around BOIs, the present review is not exhaustive. For example, we focused on BOIs induced in presence of visual information, excluding therefore the somatic versions of BOIs and similar body illusions induced with auditory cues (Tajadura-Jiménez et al., 2012). Nevertheless, similar principles to those proposed here have been revealed: e.g., temporal constraints in visuotactile integration (Ehrsson et al., 2005a) and negligible effects of tactile quality (White et al., 2010). In addition, we did not address the important contribution of interoceptive signals in the sense of body ownership (Seth, 2013), although this has recently shown to influence different aspects of BOIs (Crucianelli et al., 2013; Lloyd et al., 2013; van Stralen et al., 2014).

Moreover, our theoretical proposals based on computational principles that combine and integrate exteroceptive and proprioceptive signals for explaining the induction of BOIs, do not imply that the emergence of the sense of body ownership relies on a strictly deterministic computation. For example, interoceptive signals and emotional states are fundamental in our sense of embodiment (Carruthers, 2008; Seth, 2013). Nevertheless, we consider that formulating and experimentally validating computational models, even if these are approximate and not exhaustive, can be a first step toward deciphering the mechanisms underlying BOIs. These approaches can be extended to include further contributions and to account for a more holistic view of our own body-perception.

Beside the necessity for experimental validation of such theoretical models, future studies are needed to investigate whether and how the contribution of spatial, temporal, and semantic characteristics of crossmodal stimuli changes once BOIs are experienced. That is, while we discussed the experimental principles in the context of inducing the illusions, there is the possibility that these spatial, temporal, and semantic constraints are adapted after the onset of the illusion—a possibility discussed above as part of the causal model. The study of Hohwy and Paton (2010) points certainly in this direction: once the rubber hand illusion was induced with a standard paradigm, the illusion could be sustained even when suddenly introducing semantic or spatiotemporal violations in the crossmodal stimuli. The result of this intervention was the elicitation of unusual experiences such as tactile sensations originating from a stroked cardboard, or supernatural tactile sensations generated by an elevated finger that was not in contact with the rubber hand. Similarly, in the study of Kilteni et al. (2012) the gradual introduction of semantic violations in terms of body proportions did not abolish but sustained the ownership illusion toward a very long fake arm. The same question could be also applied to the temporal aspects of visuotactile integration; for example, does the illusion persist if the administered visuotactile stimuli progressively change from synchronous to asynchronous? Indeed, there is evidence suggesting that a full BOI triggered by seeing a realistic virtual body in spatial coincidence with the physical body, could be sustained during asynchronous visuotactile stimulation (Maselli and Slater, 2013).

Last but not least, the present review leaves the question of why somatoparaphrenic patients perceive their body parts as not belonging to themselves, unanswered. Although outside the scope of the present review, recent studies have started to explore the induction of BOIs with somatoparaphrenic patients (Jenkinson et al., 2013; van Stralen et al., 2013; Bolognini et al., 2014). Investigating whether or not the principles on multimodal triggers and semantic information mentioned here for healthy subjects, apply also for these patients, could provide a possible strategy to grasp the link between delusions and illusions of body ownership.

### Conflict of interest statement

The authors declare that the research was conducted in the absence of any commercial or financial relationships that could be construed as a potential conflict of interest.

## Footnotes

^1^The probability distribution functions in Equation (2) can be for example approximated as normal distributions in the correspondent metric.
